# Data on medicinal plants used by herbalists for boosting immunity in people living with HIV/AIDS in Uganda

**DOI:** 10.1016/j.dib.2019.105097

**Published:** 2020-01-08

**Authors:** Godwin Anywar, Esezah Kakudidi, Robert Byamukama, Jackson Mukonzo, Andreas Schubert, Hannington Oryem-Origa

**Affiliations:** a Department of Plant Sciences, Microbiology & Biotechnology, College of Natural Sciences, Makerere University, P.O.Box 7062, Kampala, Uganda; b Department of Chemistry, College of Natural Sciences, Makerere University, P.O.Box 7062, Kampala, Uganda; c Department of Pharmacology & Therapeutics, College of Health Sciences, Makerere University, P.O.Box 7062, Kampala, Uganda; d Fraunhofer Institute for Cell Therapy & Immunology (IZI), Perlickstraße 104103, Leipzig, Germany

**Keywords:** Medicinal plants, Immunostimulants, Immunity, Traditional medicine practitioners, Herbalists, HIV/AIDS

## Abstract

This Data in Brief article provides supplementary information to or earlier Ethnobotanical survey on medicinal plants used by traditional medicine practitioners to boost the immune system in people living with HIV/AIDS in Uganda [1]. We identified 71 medicinal plant species from 37 families and 64 genera. The data were analysed using descriptive statistics such as frequencies and percentages. Most of the plant species used were trees (27) and herbs (25) from the Fabaceae (15.7%) Asteraceae Phyllanthaceae (8.6%), Rubiaceae (5.7%) and Rubiaceae (5.7%) families. Additionally, we conducted a detailed literature review of the documented species to justify their use as immunostimulants. This data is derived from a larger survey to document the use of medicinal plant species in treating opportunistic infections in Uganda by Anywar et al. [2].

**Table:** Specifications Table

Subject	Plant science, Biology, Pharmacology
Specific subject area	Ethnobotany, Ethnopharmacology
Type of data	Table
How data were acquired	Ethnobotanical survey, Microsoft Excel 2011.
Data format	Raw, analysed, graphs
Parameters for data collection	Herbalists who had at least a 5 years experience in treating people living with HIV/AIDS
Description of data collection	An Ethnobotanical survey was conducted in eight districts in Uganda. The documented medicinal plants were checked against what is already known about them with regards to improving immunity from the literature
Data source location	Makerere University Kampala/Town/Region: Country: Uganda Luweero District 0.8271° N, 32.6277° E, Rakai District 0.7069° S, 31.5370° E Bushenyi District 0.4871° S, 30.2051° E Iganga District 0.6600° N, 33.4832° E Mbale District 1.0344° N, 34.1977° E Kabong District 3.5126° N, 33.9750° E Dokolo District 1.9636° N, 33.0339° E Arua District 2.9960° N, 31.1710° E
Data accessibility	Raw data was deposited in the Mendeley repository as Data, v1, 2019. DOI: 10.17632/z8sg9yj4x3.1 https://data.mendeley.com/datasets/z8sg9yj4x3/1
Related research article	G. Anywar, E. Kakudidi, R. Byamukama, J. Mukonzo, A. Schubert, H. Oryem-Origa Medicinal plants used by traditional medicine practitioners to boost the immune system in people living with HIV/AIDS in Uganda Journal: European Journal of Integrative Medicine (2029) 101011. DOI, https://doi.org/10.1016/j.eujim.2019.101011 [1]

**Table:** 

**Value of the Data** • These data point to the fact that several plant species are used by herbalists to boost immunity in people living with HIV/AIDS. • These data can be of benefit to other researchers and policy makers. • These data can be useful in understanding the use patterns and dynamics of herbal medicines and antiretroviral drugs among people living with HIV/AIDS. • The data provide a basis for further investigation of these plant species as potential drug candidates for modulating the immune system in people who are immunocompromised.

## 1 Data

**Fig. 1:**
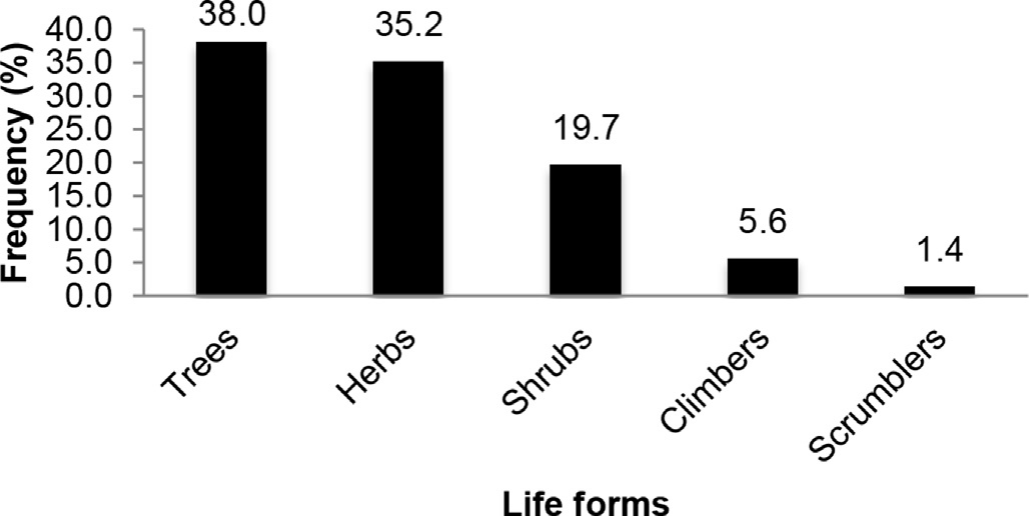
Life forms of medicinal plant species used for boosting the immune system in people living with HIV/AIDS.

**Fig. 2:**
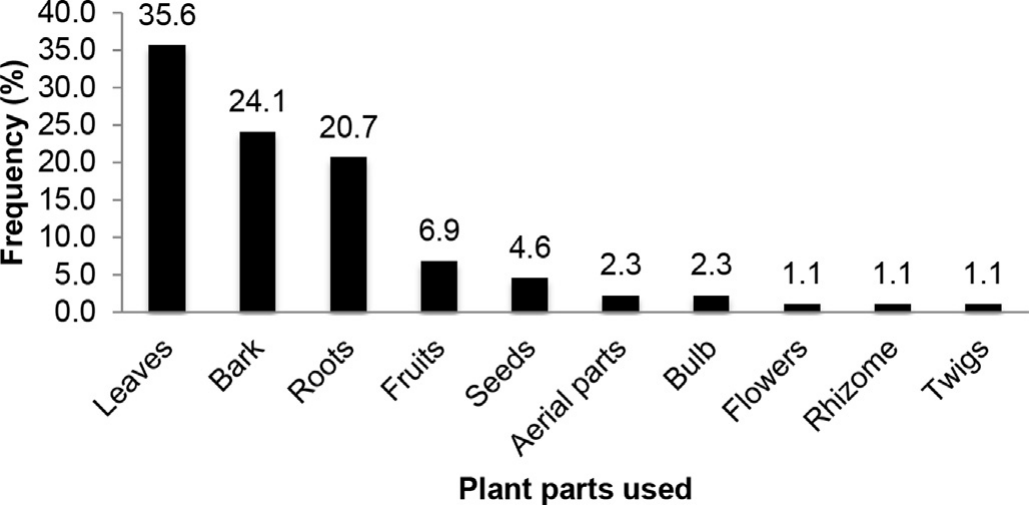
Parts of medicinal plants used for boosting immunity in people living with HIV/AIDS.

**Fig. 3:**
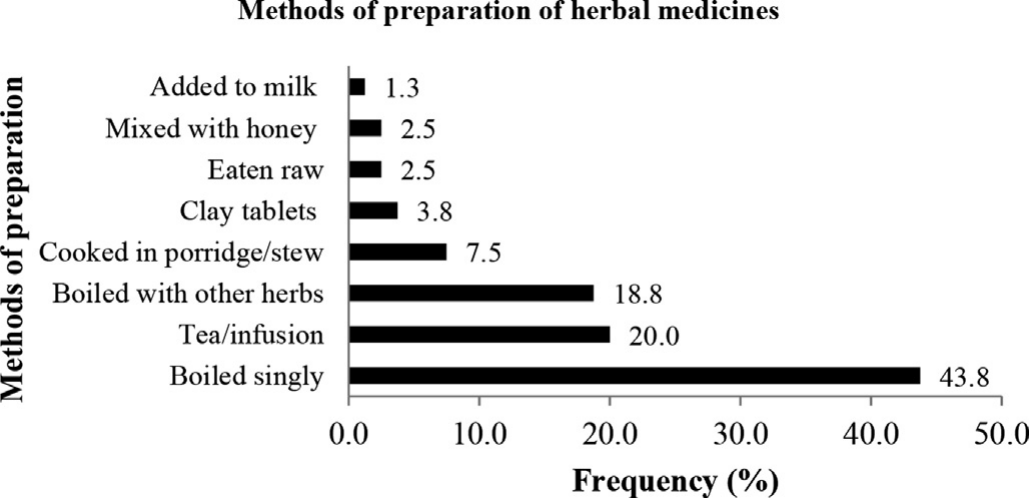
Methods of preparation of medicinal plants used for boosting immunity in people living with HIV/AIDS.

This section consists of analysed data on medicinal plant species used by herbalists for boosting immunity in people living with HIV/AIDS in Uganda. The raw data files were deposited in the Mendeley data repository DOI: 10.17632/z8sg9yj4x3.1 https://data.mendeley.com/datasets/z8sg9yj4x3/1 [2,3]. Information on the life forms of the medicinal plant species used in presented in Fig. 1, whereas the parts of the plant species used are shown in Fig. 2. In Fig. 3, the methods used for preparing and administering the respective herbal medicines are given.

## 2 Experimental design, materials, and methods

### 2.1 Ethical considerations

Ethical approval was obtained from the Higher Degrees Research and Ethics Committee of the School of Biomedical Sciences, College of Health Sciences, Makerere University, and the Uganda National Council of Science and Technology (UNCST). Written Prior Informed Consent (PIC) was also obtained from the TMP before interviewing the herbalists.

### 2.2 Ethnobotanical survey

An ethnobotanical survey was conducted on 90 TMP across the country, between March and September 2017. Different local languages were spoken in the selected districts surveyed. Only TMP who had experience of at least five years and were members of local herbalists’ associations in their districts were selected. This was done to minimise chances of dealing with quacks. Semi-structured interviews were conducted using questionnaires to gather the requisite information. Field guides and interpreters were used to help in locating the TMP and offering translation services [3].

### 2.3 Voucher specimen collection and identification

Collection of plant specimens: Field excursions with the TMP were conducted to collect voucher specimens of the plant species following standard procedures described in Martin [4]. The plant specimens were deposited at the Makerere University Herbarium for identification and classified according to the Kew database at http://www.theplantlist.org accessed on 4thJanuary-March 2018 at 18:09 EAT. The plant families were checked against the Angiosperm Phylogeny Group IV.

### 2.4 Data analysis

Ethnobotanical data obtained were analysed and presented using descriptive statistics such as percentage frequencies. Fig. 1 represents the life forms of the medicinal plant species used to boost immunity among people living with HIV/AIDS in Uganda. On the other hand, Fig. 2 shows the plant parts of the medicinal plant species used to boost immunity among people living with HIV/AIDS in Uganda, whereas Fig. 3 shows the methods of preparation and administration the medicinal plant species used to boost immunity among people living with HIV/AIDS in Uganda.
